# Antibiotic resistance pattern of microorganisms causing urinary tract infection: a 10-year comparative analysis in a tertiary care hospital of Bangladesh

**DOI:** 10.1186/s13756-022-01197-6

**Published:** 2022-12-10

**Authors:** Md. Mahabubul Islam Majumder, Ashrafur Rahaman Mahadi, Tareq Ahmed, Mostaque Ahmed, Mohammad Nazim Uddin, Md. Zahirul Alam

**Affiliations:** 1 Department of Medicine, Central Medical College, Cumilla, Bangladesh; 2 Central Medical College, Cumilla, Bangladesh; 3 Cumilla Medical College, Cumilla, Bangladesh; 4 Department of Pediatrics, Central Medical College, Cumilla, Bangladesh

**Keywords:** UTI, Antimicrobials, Antibiotics resistance, Bangladesh, Hospitals

## Abstract

**Background:**

Antimicrobial resistance is an emerging concern globally in recent years. Management of common infectious diseases like urinary tract infection (UTI) has become challenging. In this context, the purpose of this study is to compare the shifting trends in bacteriology and antibiotic resistance pattern among uropathogens to similar studies conducted at various times in Bangladesh.

**Methods:**

It was a cross-sectional study conducted at the CUMILLA MEDICAL COLLEGE HOSPITAL’S MEDICINE DEPARTMENT in three phases (2011, 2016, 2021. Patients who visited the outpatient and inpatient departments of the study center with symptoms suggestive of a urinary tract infection were undergone urine culture. Those who yielded positive growth in urne culture were finally included in the study.

**Results:**

*Escherichia coli* (62% in 2021, 86% in 2016 and 76% in 2011) and *Klebsiella species* (11% in 2021, 10% in 2016 and 11% in 2011) were the most frequently isolated bacteria. Overall, in Gram-negative organisms, resistance was almost > 50% to all the tested antibiotics. Very high frequency of resistance ranging from 66.67 to 93.75% to cotrimoxazole, ciprofloxacin, cefuroxime, cephradine, amoxicillin and nalidixic acid, moderately high resistance to ceftriaxone (64.52%) and gentamicin (53.13%) and low resistance to nitrofurantoin (25.38%) were shown by the most commonly isolated organisms. Resistance to common antibiotics has been significantly increased over time in the isolated orgnaisms, especially in carbapenem and aminoglycoside group.

**Conclusion:**

Resistance of uropathogens against conventional antibiotics used to treat UTI is high and the proportion has been increased over time. The situation might be grave in upcoming years if rational consumption of antibiotics is not warranted.

## Background

Urinary tract infection (UTI) is a common bacterial infection that affects 150 million people globally each year and is a major public health concern in terms of morbidity and financial cost [1]. UTI is defined as a medical condition characterized by the presence of pathogenic microorganisms in the urine, bladder, urethra, kidney, and prostate [2]. It is the second most prevalent bacterial infection, affecting people of all ages all over the world [3]. Globally, an estimated 50% of women will experience a UTI at least once throughout their lifetime, and UTIs are most prevalent among individuals aged 16–64 years [4].

Urinary tract infections can be caused by either community or hospital-acquired pathogens. *Escherichia coli, Klebsiella pneumoniae, Proteus mirabilis, Staphylococcus saprophyticus*, or *Enterococcus faecalis* induce community-based infections, whereas *Escherichia coli, Pseudomonas aeruginosa, Proteus, Enterobacter, Serratia, or Enterococcus faecalis* cause nosocomial infections [5].

The presence of urinary catheters and vaginal intercourse are factors that promote exposure to uropathogens, indices of host sensitivity to the disease include age, gender; and the existence of underlying disorders affecting the urinary tract. Bacterial features enhance the agent’s capacity to be spread, produce infection, and cause disease [6]. The prevalence of UTI is higher in women than in men, which might be attributable to anatomical predisposition or other host variables. Most UTIs in females are influenced by vaginal colonization with uropathogens, and sexual activity, pregnancy, and obstruction are other variables leading to the rising occurrence of UTI in female [7, 8].

In recent years, emerging antimicrobial resistance against common uropathogens has become a major challenge for management of UTI. This problem is grave in lower and middle income countries where indiscriminate and irrational use of antimicrobials is a common practice which accelerates the resistance of these molecules [9]. However, the prevalence and pattern of antimicrobial resistance and susceptibility of uropathogens are determined by a variety of factors and are constantly changing. In this context, continuous monitoring of the susceptibility pattern is extremely important for choice of treatment modality [10].

Increased resistance of urinary pathogens to frequently used antibiotics has been recorded in the eastern region of Nepal, India, and Bangladesh [11, 12]. Because the proportion of UTI-causing organisms and their antibiotic resistance and sensitivity patterns change by region, antibiotic resistance and sensitivity patterns must be studied often. Additionally, the causative agents and degree of resistance to the most frequently prescribed medications used to treat UTIs may have evolved over time. Thus, it is important for public health to monitor the local prevalence of uropathogens and their susceptibility profile on a continuous and periodic basis in order to encourage the optimal use of conventional antibiotics [6].

The present investigation was carried out to determine the recent status of prevalence of bacterial pathogens and their antimicrobial susceptibility in UTI patients. Also, this study will help us to determine the antibiotics resistance pattern at a tertiary hospital in Bangladesh and to compare it to a prior study conducted in the same pattern in 2011 and 2016 [13, 14]. It will be helpful for awareness and choice of empirical antibiotics use in UTI in this tertiary level hospital and country level.

## Methods

### Study design and setting

It was a cross sectional study conducted in the DEPARTMENT OF MEDICINE OF CUMILLA MEDICAL COLLEGE HOSPITAL, BANGLADESH during the period of June 2011–June 2021. Data were collected in three phases with 5 years interval in 2011, 2016 and 2021.

### Patients

All the patients aged above 12 years visiting the outpatient and inpatient department of the study center with a clinically suspected diagnosis of UTI (dysuria, frequency, fever and pain in lower abdomen) were considered as the study population and sent for urine culture and sensitivity test. However, patients with active menstruation, PID, tubo-ovarian disease, appendicitis, colitis, epididymitis, orchitis identified clinically or by examinations were excluded from this research. Patients already taking antibiotics were ceased from taking them for 48 h before participating in this study. Finally, patients with proven UTI by positive growth of microorganism in urine sample were included in the study. Positive culture was defined as isolation of ≥ 10^5^ colony-forming units/mL in a freshly voided midstream urine specimen.

### Laboratory procedure

Clean voided mid-stream urine (MSU) specimens were collected in sterile tubes from UTI suspect patients and transferred to the laboratory within 2 h of collection. Contamination was controlled by providing clear instructions on how to collect the sample properly. Catheters were utilized when the patient was unable to deliver a urine sample.

Using a calibrated inoculating loop with a capacity of 0.001 ml, urine from each patient was inoculated onto cysteine-lactose-electrolyte deficient agar CLED/ (Oxoid, Basingstoke, Hampshire, England) plates. The inoculation plates were aerobically incubated for 24–48 h at 37 °C. If growth observed, Plates with a colony count of ≥ 10^5^ cfu/ml were considered significant bacteriuria [15]. Then sub-cultured to Mac-Conkey agar (Oxoid, Basingstoke, Hampshire, England) and 5% sheep blood agar (Oxoid, Basingstoke, Hampshire, England) [15]. Gram stain and biochemical tests were used to characterize/identify bacterial isolates; for Gram-positive bacteria, catalase, novobiocin disk, and coagulase tests were performed; for Gram-negative bacteria, triple sugar iron agar test, indole motility test, citrate agar test, lysine decarboxylase agar test, urea agar test, and oxidase tests were performed.

On Muller Hinton agar, an antimicrobial susceptibility test was performed using the Kirby-Bauer disc diffusion technique according to Clinical Laboratory Standards Institute (CLSI) standards [16]. A suspension of 3–5 colonies of freshly grown test organism was introduced, corresponding to 0.5 McFarland standards. By spinning the swab with the suspension, the surface of the Muller-Hinton agar was thoroughly covered. After allowing the plates to dry for 3–5 min, the discs were equally spread on the inoculation plate with sterile forceps and incubated at 37 °C for 18–24 h. A ruler was used to measure the diameter of the zone of inhibition surrounding the disc. Based on the CLSI 2018 guidelines, the results were classified [16]. The following routinely used antimicrobials were tested: ampicillin [10 µg], gentamycin [10 µg], amoxicillin and clavulanic acid (amox-clav) [20/10 µg], cefoxitin [30 µg], cefotaxime [30 µg], ciprofloxacin [5 µg]; meropenem [10 µg], cotrimoxazole [trimethoprim, 1.25/sulfamethoxazole, 23.75 µg], ceftazidime [30 µg], chloramphenicol [30 µg], tetracycline [30 µg], nitrofurantoin [300 µg], and erythromycin [15 µg] [16].

### Statistical analysis

Data entry and analysis were done by using SPSS version 25.0. Descriptive statistics were used to summarize socio-demographic data, bacterial profile and susceptibility patterns of isolates. The results are summarized and presented by age groups, sex with other demographic variables and isolation types. The prevalence rate of the isolates, frequency, susceptibility and resistance patterns and other descriptive statistics were computed. Chi-square test was used to compare antibiotic resistance pattern in three phases. *P*-value < 0.05 was considered as statistically significant.

### Ethical review

The ethical permission received from the ethics review committee of CUMILLA MEDICAL COLLEGE. Prior to data collection, patients were told about the project and consented, and anonymity was maintained throughout the study by removing their names and other personal identifiers. Confidentiality was strictly maintained during data processing and report writing.

## Results

A total of 3521 patients (2312 patients in 2021, 658 patients in 2016 and 551 patients in 2011) were screened and undergone urine culture test. Among their urine sample, a total of 995 (28.3%) yielded growth of microorganisms. Growth positive rate was 28.8% (n = 666) in 2021, 30.1% (n = 198) in 2016 and 23.8 (n = 131) in 2011. The sociodemographic and clinical characteristics of the patients whose urine sample yielded growth are described in Table 1. Majority of the patients of all the included years were female and aged between 18 and 40 years. Almost two-third of the patients hailed from urban areas and belonged to middle class families. Similar proportion of patients were sexually active. Dysuria, urgency, fever and abdominal pain were the most common clinical presentation among the patients confirmed with UTI (Table 1).

**Table 1:** Sociodemographic and clinical characteristics of the patients

Characteristics	2021	2016	2011	*p*-value
*Age*				
< 18	107 (16.1)	29 (14.6)	19 (14.5)	0.142
18–40	280 (42.0)	87 (43.9)	55 (42.0)	
41–60	181 (27.2)	46 (23.2)	31 (23.7)	
> 60	98 (14.7)	36 (18.2)	26 (19.8)	
*Sex*				
Female	480 (72.1)	123 (62.1)	96 (73.3)	0.089
Male	186 (27.9)	75 (37.9)	35 (26.7)	
*Residence*				
Urban	219 (32.9)	58 (29.3)	41 (31.3)	0.214
Rural	447 (67.1)	140 (70.7)	90 (68.7)	
*Education*				
No education	182 (27.3)	51 (25.8)	34 (26.0)	0.078
Up to secondary	279 (41.9)	78 (39.4)	59 (45.0)	
Higher	205 (30.8)	69 (34.8)	38 (29.0)	
*Economic status*				
Lower class	137 (20.6)	21 (10.6)	21 (16.0)	0.253
Middle class	459 (68.9)	113 (57.1)	88 (67.2)	
Higher class	70 (10.5)	64 (32.3)	22 (16.8)	
Sexual activity				
Active	452 (67.9)	126 (63.6)	89 (67.9)	0.122
Not active	386 (58.0)	72 (36.4)	42 (32.1)	
*Co-morbid condition*				
DM	68 (10.2)	21 (10.6)	11 (8.4)	0.221
HTN	36 (5.4)	14 (7.1)	8 (6.1)	0.135
IHD	21 (3.2)	7 (3.5)	4 (3.1)	0.387
Others	34 (5.1)	6 (3.0)	2 (1.5)	0.085
*Clinical feature*		(0.0)		
Dysuria	483 (72.5)	138 (69.7)	102 (77.9)	0.147
Urgency	454 (68.2)	157 (79.3)	87 (66.4)	0.098
Fever	512 (76.9)	142 (71.7)	98 (74.8)	0.096
Abdominal pain	446 (67.0)	141 (71.2)	79 (60.3)	0.097

*Escherichia coli* and *Klebsiella species* were the most frequently isolated gram-negative bacteria from the UTI patients’ urine sample over the study period. Other urinary tract bacteria contributing significantly to the burden of UTIs (gram-negative) included *Pseudomonas species*, *Proteus species*, *Enterobacter species*, *Acinetobacter species*, and *Salmonella typhi*. On the other hand, the major gram-positive pathogens include *Staphylococcus aureus*, *Enterococcus species*, and *Streptococcus species* (Table 2).

**Table 2:** Distribution of significant uropathogenic microorganisms among patients with urinary tract infection

Organism	2021*	2016	2011
Gram negative			
*Escherichia coli*	410 (61.6)	171 (86.0)	98 (75.5)
*Klebsiella spp.*	73 (11.0)	17 (9.6)	14 (10.7)
*Pseudomonas spp.*	31 (4.7)	0 (0.0)	2 (1.5)
*Proteus spp.*	22 (3.3)	0 (0.0)	2 (1.5)
*Enterobacter spp.*	4 (0.6)	0 (0.0)	0 (0.0)
*Acinetobacter spp.*	1 (0.2)	0 (0.0)	0 (0.0)
*Salmonella typhi*	1 (0.2)	0 (0.0)	0 (0.0)
Gram positive			
*Staphylococcus aureus*	45 (6.8)	0 (0.0)	4 (3.0)
*Enterococcus spp.*	45 (6.8)	10 (5.0)	8 (6.0)
*Streptococcus spp.*	32 (4.8)	0 (0.0)	0 (0.0)
Fungus			
*Candida spp*.	15 (2.3)	0 (0.0)	3 (2.3)

*Percentage is > 100% as some of the samples yielded growth of multiple organisms simultaneously

The antibiotic resistance of the frequently isolated uropathogens is presented in Table 3. Overall, in Gram-negative organisms, resistance was almost > 50% to all the tested antibiotics. However, a low level of resistance has been observed for Gram-negative agents against meropenem and imipenem. In contrast, in the case of *K. pneumoniae*, showed very less sensitivity to Azithromycin (93.75%) and almost similar pattern has been observed for Azithromycin (93.75/93.33%) in *Pseudomonas* spp. and *Proteus* spp. (Table 3) *Pseudomonas* spp., *Proteus* spp., And *Staphylococcus aureus* showed 100% resistance against Ampicillin.

In our study, the highest resistance (> 70%) was observed against *E. coli Klebsiella spp*., and *Proteus* spp. to almost all the tested antibiotics except carbapenem, which were in the range of 1.47–22.58%. A similar pattern has been observed for other Gram-negative uropathogens. The lowest observed resistance for *Klebsiella* spp. and *Proteus* spp. was no resistance against meropenem and imipenem.

Very high frequency of resistance ranging from 66.67 to 93.75% to cotrimoxazole, ciprofloxacin, cefuroxime, cephradine, amoxicillin and nalidixic acid, moderately high resistance to ceftriaxone (64.52%) and gentamicin (53.13%) and low resistance to nitrofurantoin (25.38%) were shown by *E. coli.* Similarly, *Staphylococcus aureus, Streptococcus spp.* and *Enterococcus* spp. showed low resistance (26.7, 19.23 and 25.58%) to nitrofurantoin, but moderately high against gentamycin, cefuroxime and ceftriaxone. On the other hand, *Staphylococcus aureus* shows 100% resistance against Ampicilin.

**Table 3:** Antibiotic resistance pattern of different organisms isolated from the urine specimen of the patients (combined of all years, n = 995)

Antibiotic	*E.coli*	*Klebsiella spp.*	*Proteus spp.*	*Pseudomonas spp.*	*Staphylococcus aureus*	*Streptococcus spp.*	*Enterococcus spp.*
	R (%)	R (%)	R (%)	R (%)	R (%)	R (%)	R (%)
*Carbapenem*							
Imipenem	3.92	0.00	0.00	22.58	0.00	NA	0.00
Meropenem	1.47	0.00	0.00	17.24	16.28	9.68	19.51
*Cephalosporin*							
Cephradine	83.33	NA	NA	75.00	41.46	18.75	54.05
Cefotaxime	61.75	50.00	7.69	52.63	42.50	10.00	50.00
Ceftazidime	50.00	41.10	9.52	60.00	50.00	50.00	75.00
Cefuroxime	65.77	54.17	18.18	87.10	42.86	12.90	59.09
Ceftriaxone	58.62	49.32	0.00	64.52	42.22	12.90	60.00
Cefixime	77.94	58.90	18.18	87.10	93.18	31.25	70.45
Cefepime	50.51	43.84	4.76	41.94	33.33	50.00	0.00
*Quinolone*							
Ciprofloxacin	51.72	27.40	36.36	66.67	53.33	35.48	63.64
Levofloxacin	43.36	15.49	36.36	55.17	47.73	29.03	45.45
Nalidexic Acid	83.38	50.00	68.42	85.71	93.33	100.00	87.50
*Aminoglycoside*							
Amikacin	5.91	1.37	4.55	19.35	13.64	78.13	68.18
Gentamycin	20.79	22.22	23.81	35.48	29.55	53.13	37.78
Penicillin							
Amoxiclav	35.38	22.22	27.27	83.33	11.11	6.25	8.89
Amoxycillin	NA	NA	NA	NA	NA	12.50	5.26
Ampicilin	62.50	66.67	100.00	100.00	100.00	14.29	0.00
*Macrolides*							
Azithromycin	89.49	93.75	93.75	93.33	79.07	67.74	92.86
Erythromycin	NA	NA	NA	NA	80.00	75.00	87.50
Clindamycin	NA	NA	NA	NA	31.82	35.48	62.79
*Others*							
Nitrofurantion	25.38	79.41	84.21	85.71	26.67	19.23	25.58
Cotrimoxazole	52.79	36.76	77.27	89.66	53.33	86.21	85.71
Piperacillin/ Tazobactum	11.55	9.72	0.00	22.58	NA	NA	50.00
Vancomycin	2.82	7.14	0.00	0.00	0.00	0.00	0.00
FusidicAcid	NA	NA	NA	NA	11.36	91.67	88.57
Doxycycline	46.00	53.42	76.19	80.00	2.27	62.50	75.56
Linezolid	1.35	0.00	0.00	0.00	0.00	0.00	2.33
Tigecycline	0.00	6.82	4.41	9.52	0.00	3.13	0.00

Resistance to different classes of antibiotics has been significantly increased over time in the isolated orgnaisms, especially in carbapenem and aminoglycoside group. Resistance to both imipenem and meropenem was almost 4% in 2021 while it was almost nil in previous years. Similar trend was observed in case of amikacin, gentamycin, amoxyclav and nitrofurantoin (Table 4).

**Table 4:** Comparison of antibiotic resistance pattern of UTI causing organism during different point of time

Antibiotics	Resistance rate (%)			*p*-value
	2021	2016	2011	
Carbapenem				
Imipenem	4.2	0.5	0.0	0.007
Meropenem	3.4	0.0	2.0	0.021
Cephalosporin				
Cephradine	81.6	65.0	63.0	0.782
Cefotaxime	61.6	58.0	61.0	0.241
Cefuroxime	64.3	55.0	63.0	0.638
Ceftriaxone	58.8	55.0	60.0	0.078
Cefixime	78.9	60.0	70.0	0.254
Quinolone				
Nalidexic acid	86.5	55.0	75.0	0.440
Ciprofloxacin	52.8	48.0	66.0	0.084
Aminoglycosides				
Amikacin	6.2	1.0	2.0	0.017
Gentamycin	23.7	10.0	14.0	0.043
Penicillin				
Amoxyclav	77.4	76.0	24.0	0.036
Amoxycillin	NA	86.0	87.0	0.990
Others				
Nitrofurantoin	31.2	12.0	9.0	0.002
Cotrimoxazole	58.7	47.0	62.0	0.089

## Discussion

Because of the high prevalence of infection in the community and hospital setting, urinary tract infections have imposed a significant financial burden on the health system [17]. Effective treatment of patients with bacterial urinary tract infections is often dependent on pathogen identification and antibiotic selection based on ongoing surveillance of the antimicrobial susceptibility pattern of urinary tract pathogens in specific regions [18]. The current study’s findings provide light on antimicrobial resistance patterns in Bangladesh, a country with sparse antimicrobial resistance surveillance data.

In this study, most of the uropathogens were isolated from female patients. Several predisposing factors might contribute to the higher prevalence of UTIs among women [19]. It is well recognized that UTI is more prevalent in female than in male, and our data corroborate this generalization and correspond with a previous study conducted by Deshpande et al. [20]. Similarly, our observation on the prevalence of uropathogens is consistent with other prior reports [21]. We found that women of reproductive age are most susceptible group for UTI. Vaginal colonization with pathogens, and sexual activity are identified as risk factors of UTI in women of this age group in previous studies[7, 8] Besides, prevalence of UTI was also high in post-menopausal women. This phenomenon might be a result of genito-urinary atrophy and vaginal prolapse after menopause that alters the vaginal pH, decreasing the normal vaginal flora. This condition allows for Gram-negative bacteria to grow as uropathogens [22].

Antibiotic resistance is a major concern when it comes to common bacterial infections, including UTI. Antimicrobial drugs such as amoxicillin, cotrimoxazole, cephradin, nalidixic acid, ciprofloxacin, and azithromycin are still used in many underdeveloped and developing countries, including Bangladesh, to treat a variety of gram-positive and gram-negative bacterial infections, including UTI. Unfortunately, all of these drugs were shown to have an unacceptable spectrum of antibacterial activity against uropathogens identified in our research. This outcome is concerning in terms of the availability of effective therapeutic options in the treatment of UTI and should be of major concern to treating physicians. Ciprofloxacin was originally thought to be the treatment of choice for both uncomplicated and complicated UTI, however owing to a lack of sensible usage, this broad-spectrum drug has completely lost its efficacy not just in UTI but also in other frequent infections. A similar image may be seen with first, second, and third generation cephalosporins. It is possible to hypothesize that a few cases of extended spectrum beta-lactamase (ESBL)-producing uropathogens, particularly Gram-negative isolates, that could not be isolated in the current analysis due to limitations are considered to be responsible for resistance to a variety generation of cephalosporin.

*E. coli* demonstrated the greatest multidrug resistance among the identified uropathogens, with more than 70% of the isolates resistant to more than five antibiotic classes tested. This is comparable to a research done in a tertiary hospital in Pakistan [22].

Nitrofurantoin was shown to be a relatively effective agent among all antimicrobials used to treat practically all uropathogens in the current setting, and similar findings have been reported in other investigations [21, 23].

This is really reason for optimism, especially for uncomplicated UTI and prophylaxis, given the continuously declining susceptibility of most of the considerably less expensive oral anti-UTI medications. As a result, nitrofurantoin can be considered as a first-line, cost-effective, and cost-effective oral treatment in UTI.

There was a considerable reduction in sensitivity pattern for imipenem, ceftriaxone, and amoxiclav in the years 2021, 2016, and 2011, presumably due to random usage of these antibiotics in the previous few years with inadequate dosage and duration. Antibiotic resistance in uropathogens has now become a public health problem in Bangladesh [24].

This steep increases in antibiotic resistance might be attributable to the irrational use of antibiotics for the treatment of various diseases without culture sensitivity test [25].This perilous state is the major cause of MDR infection in urinary tract infections. The resistance to cotrimoxazole was > 60% in all isolated Gram-negative organisms. Cotrimoxazole is the first-line empirical antibiotic recommended by the European Urology Association and the Infectious Disease Society of America (IDSA) for the treatment of uncomplicated community-acquired urinary tract infections (UTIs) where locally reported resistance percentages against uropathogens range from 10 to 20% [11]. Regrettably, our study found the highest resistance rates to cotrimoxazole. This development in resistance may be a result of indiscriminate antibiotic usage and the medicines’ availability without a physician’s prescription.

*Pseudomonas spp*. is the most well-known gram-negative isolate responsible for hospital-acquired urinary tract infections, and traditional antimicrobials are typically ineffective against Pseudomonas infections[7]. While nitrofurantoin has a high susceptibility to *Pseudomonas* UTI, it is only advised for uncomplicated UTI or prophylaxis. Although important antibiotics such as carbapenems are being used to treat *Pseudomonas* infections, we propose that their usage be limited to exceptional cases in order to retain their long-term efficacy.

Overall, UTI with *E. coli* was significantly increased in the year 2021. This study showed a steady increase in resistance to all studied antibiotics. Imipenem, meropenem, tazobactam, amikacin and nitrofurantoin are increasing their sensitivity pattern in this study. Comparative study of 2021, 2016 and 2011 shows significant resistance to different classes of antibiotics has been significantly increased over time in the study patients, especially in carbapenem and aminoglycoside group. Resistance to both imipenem and meropenem was almost 4% in 2021 while it was almost nil in previous years. Similar trend was observed in case of amikacin, gentamycin, amoxiclav and nitrofurantoin.

According to this Infectious Diseases Society of America (IDSA) guideline, the majority of antibiotics used in our study should not be administered empirically for acute UTI, and our current standard treatment guidelines for UTI are inadequate, requiring a large-scale investigation.

## Limitations

As our study is based on a cross sectional dataset during different point of time, it has several limitations. Although the health institution provides high-quality clinical and laboratory services and keeps correct paperwork, one cannot guarantee that the data quality checks adhere to the highest requirements of a good research. Although the sample collection, microbiological analysis, and report updates followed standard protocols, they were almost certainly carried out by various persons during a ten-year period, resulting in inter-personnel variances in the quality of laboratory processes. Additionally, laboratory reports are manually recorded into a computerized system, which is prone to human mistake, and data entry parameters are sensitive to human deviation.

We recognize several flaws in our study due to a lack of clinical data. Though the distribution of uropathogens is somewhat consistent across settings, rising antibiotic resistance to bacteria that cause UTI is a major worry worldwide, but particularly in emerging and underdeveloped nations.

## Conclusion

Resistance to routinely used antibiotics is shown to be quite significant. This demonstrates that antibiotic resistance is a significant issue in Bangladesh. Inadequate treatment regimens, insufficient patient adherence, unregulated drug distribution and trafficking, as well as antibiotic scarcity and poor quality, can all contribute to antibiotic resistance. To that purpose, the frequency of urinary tract infections should be reduced, and the sensitivity of certain microorganisms to routinely used antibacterial drugs should be checked on a constant basis. These findings have the potential to influence local objectives, policies, and practices. We urge that big health institutions monitor and assess developing AMR patterns and trends on a regular basis in order to prioritize, plan, and execute health facility level policies and guidelines for antimicrobial stewardship.

Finally, a comprehensive survey and research on antibiotic resistance are required to analyze this disastrous national situation and develop management solutions.

## Data Availability

Data will be made available on request.
